# *Aspergillus* and Health

**DOI:** 10.3390/microorganisms10030538

**Published:** 2022-02-28

**Authors:** Raquel Sabino

**Affiliations:** 1 Reference Unit for Parasitic and Fungal Infections, Department of Infectious Diseases, National Institute of Health, 1649-016 Lisbon, Portugal; raquel.sabino@insa.min-saude.pt; Tel.: +351-217519247; 2 Instituto de Saúde Ambiental, Faculdade de Medicina, Universidade de Lisboa, 1649-028 Lisboa, Portugal

Among the filamentous fungi described as etiological agents of infection, *Aspergillus* is the most frequent agent of invasive mould disease, and it is associated with high mortality. The Special Issue “*Aspergillus* and Health 1.0” intended to emphasize the enormous relevance of this agent as potential pathogen, and with this purpose, we gathered a diverse set of studies (nine research papers and two reviews) addressing *Aspergillus* epidemiology, prevalence, diagnosis, antifungal resistance, resistance mechanisms and virulence traits. In the following lines, the main topics of this Special Issue are revised, inviting all the readers to a deeper analysis of the published manuscripts.

An effective diagnosis of aspergillosis is complex and challenging. The development and validation of diagnostic tools and laboratorial methods to increase the accuracy to detect *Aspergillus* infection are always in the scope of scientific research. Culture remains the gold standard for the laboratory diagnosis of invasive fungal infections, including aspergillosis. Aiming to attain a higher sensitivity of cultures (especially in samples contaminated with different fungal genera), Zhang et al. [1] suggested the use of Flamingo Medium as an efficient method for the isolating *A. fumigatus*, especially from tissue contaminated with fungi belonging to Mucorales order. The authors reported that, using Flamingo Medium, they were able to recover an average of 20−30% more *A. fumigatus* colonies compared to other media, and at the same time, a 95% reduction in the number of Mucorales colonies was also observed. Also with the purpose of improving the diagnosis of aspergilosis, Cerqueira et al. [2] presented a method developed for detection of *A. fumigatus* sensu stricto in respiratory samples using peptide nucleic acid-fluorescence in situ hybridization (PNA-FISH). The sensitivity of the tested probe was 100% in strains but 79% in clinical samples, with a specificity of 100% in both cases. This study showed the potential of the PNA-FISH method for *A. fumigatus* sensu stricto detection, although more developments need to be done in order to achieve higher sensitivity in clinical samples.

The genus *Aspergillus* has been subject to a large number of taxonomic studies using DNA sequence data. Molecular characterization of *Aspergillus* has been contributing to the knowledge of molecular epidemiology, to the discovery of new etiological agents and to the understanding of species distribution. Climatic and geographic conditions may be important determinants of the local prevalence and distribution of *Aspergillus* species, which contribute to a diverse epidemiology among regions. Aiming to address some of these questions, Dietel et al. [3] performed a one-year study collecting environmental samples in Tyrol, Austria. Given the high frequency of aspergillosis cases caused by *A. terreus* in Austria, the authors aimed to perceive its ecological niche, distribution and association with clinical cases. They concluded that soil from agricultural cornfields seems to be an important source of *A. terreus* and that the environmental frequency of *A. terreus* correlates with the high incidence of *A. terreus* infections in certain geographical areas. Genotypic studies were also performed and allowed the identification of three major genotypes in Tyrol, with an elevated percentage of the studied isolates resistant to several antifungals, especially to amphotericin B (AMB).

Interestingly, Fan et al. [4] also focused their study in the high rate of AMB resistance but in *A. fumigatus* isolates. Using a whole genome approach in isolates from different sources, the single nucleotide polymorphisms (SNPs) were analysed, and three divergent genetic clusters were defined. These strains belonged to three divergent genetic clusters and 90% of AMB-resistant strains were located in one of those three clusters (cluster 2). More than 60 SNPs were significantly associated with AMB resistance, which may represent targets for the study of molecular mechanisms of resistance. 

In addition, the high mortality rate in immunocompromised patients, significant pulmonary pathology is also associated with *Aspergillus*-induced allergic and asthmatic lung disease related to occupational exposure, associated with local epidemiology. Having this in mind, Sánchez Espinosa et al. [5] identified species of the genus *Aspergillus* obtained from the indoor environment of houses located in different municipalities of Havana, Cuba. The dust, walls and air of houses with history of moisture were collected and the diversity and richness of the isolated *Aspergillus* were analysed. The application of molecular methods for achieving the correct species identification within a specific *Aspergillus* section allowed the detection of numerous cryptic species and the identification of 15 species that have never been identified from the environment in Cuba. According to the authors, in addition to contributing to the knowledge of fungal biodiversity and ecology, those findings constitute an alert for the health authorities, since prolonged exposure of the inhabitants to mouldy houses can cause severe persistent asthma, among other diseases.

Azole resistance has been increasing in prevalence in *A. fumigatus* isolates due to the development of acquired resistance caused by prophylaxis/treatment with antifungals, as well as to the use of agricultural azoles and consequent acquisition of resistant isolates from environmental origin, posing new challenges in therapeutic management. In occupational environments where high fungal loads are expected, there is an increased risk of human exposure and infection, with impact on treatment success and disease outcome. Within that scope, in the study presented by Gonçalves et al. [6], 99 *A. fumigatus* isolates collected from indoor environments were screened for azole resistance and 3% of those isolates were pan-azole-resistant, bearing the TR34/L98H mutation. Resistant isolates were collected from an air sample from a diary and from disposable filtering respiratory protective devices worn by workers of a waste sorting plant. For the first time in Portugal, resistant isolates were detected from occupational environments, highlighting the importance of these local epidemiological studies on implementation of control policies that may have impact on occupational and public health. Following the subject of azole resistances in *A. fumigatus*, the review published by Melo et al. [7] discusses the emergence of azole resistance on *Aspergillus* considering the One Health Context. The authors propose that birds can play an important role in the dispersion of *Aspergillus*, and of special concern, azole-resistant strains, since avian species are involved in short and long distances travel between different types of landscapes, such as agricultural fields, where agricultural azole pesticides are used and may induce azole resistances. 

The field of research on antifungal resistances is always a hot topic for the management of *Aspergillus* infections. In the work published by Schwarz et al. [8], the in vitro interactions of isavuconazole in combination with colistin were evaluated against *A. flavus*, *A. fumigatus*, *A. nidulans*, *A. niger* and *A. terreus*. Colistin is a drug with activity against multidrug-resistant gram-negative bacteria, and this study showed that colistin enhances the in vitro activity of isavuconazole against clinical *A. nidulans* and *A. niger* isolates with high in vitro MICs to isavuconazole using the EUCAST broth microdilution checkerboard methodology. On the other hand, by using an agar diffusion assay method, the susceptibility of *A. nidulans* was not affected by isavuconazole in combination with colistin, whereas only some of the *A. niger* isolates were. These studies on possible synergies are promising since they can help to establish new management approaches. 

One of those studied species, *Aspergillus nidulans*, seems to have a remarkable association with chronic granulomatous disease patients and with mycotoxin production and these features have been raising the awareness on this species and its possible role in pathogenesis. Ye-Eun Son et al. [9] observed, by transcriptomic analysis, that the product codified by the gene *vadA* affects the mRNA expression of a variety of genes in *A. nidulans* conidia. The genes that were primarily affected in conidia were associated with trehalose biosynthesis, cell-wall integrity, stress response, and secondary metabolism. Additionally, the deletion of *vadA* led to an increase in the amount of the mycotoxin sterigmatocystin in the conidia. Data obtained in this study suggest that VadA coordinates conidia maturation, stress response, and secondary metabolism in *A. nidulans* conidia.

The inhalation of *Aspergillus* conidia and their airway deposition occurs every day. *Aspergillus* disease is characterized either by damage from the fungus itself or through an exaggerated inflammatory response of the host, depending also on the underlying immune status of the host. A high frequency of patients with chronic respiratory disease, such as interstitial pneumonia or pulmonary fibrosis, do not develop any kind of *Aspergillus* infection, despite the colonization of their airways. Thus, the study published by Kushima et al. [10] intended to determine the role of *A. fumigatus* colonization in pulmonary fibrosis by using heat-inactivated conidia for the infection of human lung epithelial cells and of murine embryo fibroblast cells in 2D and 3D cultures, analysing the process by RNA-sequencing. The authors concluded that *A. fumigatus* affects both apoptosis of epithelial cells and the growth of fibroblasts.

Other group of patients with respiratory disease are the patients with cystic fibrosis (CF), whose impaired mucociliary clearance and airways are filled with thick mucus and where the inhaled *Aspergillus* conidia are easily trapped. Susceptibility to *Aspergillus*-related lung disease in CF is reflected by clinical phenotypes ranging from persistent *Aspergillus* infection and bronchitis to allergic and airway invasive aspergillosis. Given the major importance of these patients, Margalit et al. [11] published a review on polymicrobial interactions that occur in the pulmonary tract of CF patients. The authors review the agonistic and antagonistic interactions that occur between *A. fumigatus* and pulmonary bacterial pathogens such as *Pseudomonas aeruginosa*. According to the revised studies, although bacteria may predominate in a competitive environment, *A. fumigatus* has the capacity to persist and contribute to disease.

Despite very different in their content, all the manuscripts published in this Special Issue followed the proposed conductive line on *Aspergillus* and Health and discussed the most recent insights on this area. 

As such, I would like to thank to all the authors who contributed with their excellent papers to this Special Issue and to the reviewers for their time and expertise in examining and commenting on the manuscripts. Thanks also to the staff of the *Microorganisms*’ Editorial Office for their management and organization of this Special Issue. Given the elevated participation in this issue, we have the pleasure to launch the Special Issue “*Aspergillus* and Health 2.0”, to which all are invited to contribute with prominent research.
